# Designer Self-Assemble Peptides Maximize the Therapeutic Benefits of Neural Stem Cell Transplantation for Alzheimer’s Disease via Enhancing Neuron Differentiation and Paracrine Action

**DOI:** 10.1007/s12035-014-9069-y

**Published:** 2015-01-14

**Authors:** Guo-hong Cui, Shui-jin Shao, Jia-jun Yang, Jian-ren Liu, Hai-dong Guo

**Affiliations:** 1 Department of Neurology, Shanghai No. 9 People’s Hospital, School of Medicine, Shanghai Jiaotong University, 639 Zhizaoju Road, Shanghai, 200011 China; 2 Department of Neurology, Shanghai No. 6 People’s Hospital, School of Medicine, Shanghai Jiaotong University, Shanghai, 200233 China; 3 Department of Anatomy, School of Basic Medicine, Shanghai University of Traditional Chinese Medicine, 1200 Cailun Road, Shanghai, 201203 China

**Keywords:** Alzheimer’s disease, Neural stem cell, Transplantation, Self-assembling peptide, Differentiation

## Abstract

The neuropathological hallmarks of Alzheimer’s disease (AD) include the presence of extracellular amyloid-β peptide (Aβ) in the form of amyloid plaques and neuronal loss. Neural stem cell (NSC) is being scrutinized as a promising cell replacement therapy for various neurodegenerative diseases. However, the unfavorable niche at the site of degenerative disease is hostile to the survival and differentiation of transplanted cells. Here, we undertook in vitro and in vivo works to examine whether a designer self-assemble peptide (DSP), which contains one functional domain Tyr-Ile-Gly-Ser-Arg (YIGSR) derived from laminin, promotes the survival and neuronal differentiation of NSC and behavioral improvement. We found that DSP could undergo spontaneous assembly into well-ordered nanofibers, and it not only facilitated the cell viability in normal culture condition, but also decreased the number of apoptotic cells induced by Aβ in vitro. NSC seeded in DSP showed much more neuronal differentiation than that seeded in self-assemble peptide (SP) or alone. In the AD model, NSC transplantation in DSP-treated AD rats demonstrated much more obvious cognitive rescue with restoration of learning/memory function compared with NSC transplantation in SP, NSC alone, or DSP alone treated ones. Interestingly, DSP enhanced the survival and neuronal differentiation of transplanted NSC. Apoptosis levels in the CA1 region and Aβ level in the hippocampus were significantly decreased in the group of NSC transplantation in DSP. Moreover, synaptic function, indicated by the expression of pre-synaptic protein synapsin-1, was restored and the secretion of anti-inflammatory and neurotrophic factors were increased, such as IL-10, brain-derived neurotrophic factor (BDNF), ciliary neurotrophic factor (CNTF), and insulin-like growth factor 1 (IGF-1), while the expression of pro-inflammatory factors were decreased, such as TNF-α and IL-1β. These data firstly unveiled that the biomaterial DSP can maximize the therapeutic benefits of NSC transplantation for AD through improving the survival and differentiation of transplanted stem cells and promoting the effects of neuroprotection, anti-neuroinflammatory and paracrine action. Our results may have important clinical implications for the design of future NSC-based strategies using the biomaterials for various neurodegenerative diseases including AD.

**Electronic supplementary material:**

The online version of this article (doi:10.1007/s12035-014-9069-y) contains supplementary material, which is available to authorized users.

## Introduction

Alzheimer’s disease (AD) accounts for more than 60 % of all dementias and the prevalence of AD expected to increase dramatically as the life expectancy and aging population rise worldwide [1]. AD involves a progressive decline in mental function, usually including learning and memory deficits, language difficulties, daily activities impairment, and other cognitive processes. The neuropathology is characterized by extracellular deposition of amyloid-β peptide (Aβ)-containing senile plaques, the intracellular presence of numerous neurofibrillary tangles, and selective loss of neurons [2]. The amyloid cascade hypothesis suggesting a pivotal role for Aβ in the pathogenesis of AD is the mechanism that is accepted by most researchers in this field [3]. The peptide with 40 or 42 amino acids has been shown in numerous studies to be toxic to neuron and thought to be responsible for neuronal and synaptic loss, leading to progressive cognitive decline in patients with AD [4–6]. Current approved therapies, including acetylcholinesterase inhibitors and glutamate inhibitors offer only marginal benefits and do not compensate for the massive and progressive neuronal and synaptic loss in the cortex and hippocampus [7, 8]. Besides, in animal models of AD, neurogenesis has been shown to be impaired, which resulted in a loss of neuron gradually with limited recruitment [9]. Thus, one possible treatment strategy for AD would be to prevent and/or replace neurons lost during the disease process.

Neural stem cell (NSC) has the potential for self-renewal and ismultipotent cell that is capable of generating the main cell phenotypes of the central nervous system, including neurons, astrocytes, and oligodendrocytes. Thus, it has been suggested that NSCs are capable of compensating for lost or damaged neurons in many neurological diseases. NSC transplantation has been tested as a potential treatment of neurological disorders, including traumatic brain injury, stroke, ischemia, and neurodegenerative disorders [10–12]. With respect to AD, studies have shown that NSC transplantation improved the cognitive ability of a transgenic model of AD via increasing brain-derived neurotrophic factor (BDNF) [13]. The most recent report found that NSC transplantation could not only replace the damaged or lost neurons resulting from Aβ plaque but also be helpful to establish new synaptic connectivity to improve cognitive dysfunction [14].

Although NSC transplantation is a potential approach for AD, the sustained survival and neuronal differentiation of exogenously transplanted NSCs, as well as their functional integration into host neuronal circuitry, remain a major challenge [15]. The pathological environment, especially the presence of aggregated Aβ peptide in AD brains has a negative effect on the survival, migration, and differentiation of both endogenous and exogenous NSC [16]. Aβ peptide has differential effects on the human embryonic stem cell proliferation depending on its aggregation level [17, 18]. The viability of NSC treated with Aβ_25–35_ oligomers was significantly decreased in a concentration-dependent manner [19]. Thus, development of clinically relevant and feasible strategies that can enhance proliferation, survival, neuronal differentiation, and functional integration of transplanted NSCs into neuronal networks of the host’s brain is urgently required if exogenously transplanted NSCs are to be utilized as a clinically effective therapeutic approach to neurodegenerative disorders including AD.

Attempts have been made to incorporate cells into biomaterials that might help restore a three-dimensional environment facilitating cell survival, proliferation, and differentiation. An ideal biomaterial should be fabricated from a synthetic biological material with defined constituents. Self-assembling peptide RADA16 consist of alternating hydrophilic and hydrophobic amino acids and could form a nanofiber scaffold under physiological conditions [20]. The scaffolds can support neuronal cell attachment and differentiation as well as extensive neurite outgrowth. Furthermore, they are permissive substrates for functional synapse formation between the attached neurons [21]. Materials functionalized with laminin or laminin-derived peptides have been shown to increase neurite extension [22], and influence cellular attachment, migration, proliferation, and differentiation [23, 24], which indicates that it is promising as an extracellular matrix platform for regeneration. Tyr-Ile-Gly-Ser-Arg (YIGSR) is a laminin-derived motif and was beneficial to promote cell attachment and laminin receptor binding [25]. YIGSR-functionalized matrices have been found to improve the neurite sprouting and regeneration. Moreover, matrices modified with YIGSR have shown similar or superior biologic effects to another laminin-derived bioactive peptide Ile-Lys-Val-Ala-Val (IKVAV) and laminin-coated matrices [26, 27].

Based on these findings, we further investigated the potential therapeutic effects of YIGSR-functionalized self-assemble peptide RADA16 in a rat model of AD generated by Aβ injection. The primary goal of the study is to test the potential utility of a designer self-assemble peptide (DSP) with functional domain YIGSR to promote the survival and neurogenesis of NSC transplanted into the hippocampus, and further to estimate its advantage to reduce hippocampus neuronal apoptosis and Aβ level, and to enhance the synaptic function and paracrine action, thereby facilitating the clinical use of NSC transplantation for the treatment of a number of neurodegenerative diseases including AD.

## Materials and Methods

The animals were randomly assigned and raised on a 12-h light/dark cycle at constant temperature, with free access to food and water. The study was approved by the Shanghai Ethics Committee and all animal procedures were performed in accordance with the National Institutes of Health Guide for the Care and Use of Laboratory Animals. We made every effort to minimize both the number of animals used and animal suffering.

### Designer Peptide Synthesis and Atomic Force Microscopy Observation

To get the functionalized peptide, we modified the self-assembling peptide RADA16 (Ac-RADARADARADARADA-CONH_2_) with a bioactive motif YIGSR, derived from laminin. Two glycine residues were added, between the assembling portion of the peptides and the functional motifs, for flexibility. The sequence of DSP is: Ac-(RADA)_4_GGYIGSR-CONH_2_. The DSP and RADA16 (self-assemble peptide, SP) were custom-synthesized. They were dissolved in distilled sterile water at a final concentration of 1 % (w/v) (10 mg/mL) and sonicated for 30 min before use. The structures of the peptides were observed under atomic force microscopy (AFM) using a tapping mode. Peptide from stock solutions (1 %) was diluted to a working concentration of 0.01 % (*w*/*v*) with distilled sterile water. After sonication for 30 min, 1-μL sample was loaded onto a freshly cleaved mica surface. To optimize the amount of peptide adsorbed, each aliquot was left on mica for 60 s and then washed with 100 μL of deionized water at least three times. The mica surface with the adsorbed peptide was then air-dried and imaged immediately. Images with a resolution of 512 × 512 pixels were obtained by an AFM microscope (Nanoscope-MultiMode/Dimension, Digital Instruments, Santa Barbara, CA) running in a tapping mode. Typical scanning parameters were as follows: tapping frequency 75 KHz, RMS amplitude before engage 1–1.2 V, set point 0.7–0.9 V, integral and proportional gains of 0.2–0.6 and 0.4–1.0, respectively, and scan rate of 1.51 Hz.

### Neural Stem Cell Isolation and Culture

NSCs were isolated directly from the hippocampus of postnatal day 1 male Sprague-Dawley (SD) rats. Gender of rats was determined by real-time polymerase chain reaction (RT-PCR) with rat sex-determining Region Y (SRY) primers according to the protocols published by Müller-Ehmsen et al. [28]. After being dissociated mechanically, the cells were harvested by centrifugation and resuspended in Neurobasal medium (Gibco, CA, USA) containing B-27 supplement (2 %, Gibco, USA), 20 ng/mL basic fibroblast growth factor (bFGF, PeproTech, USA), 10 ng/mL epidermal growth factor (EGF, PeproTech, USA), penicillin (50 U/mL), and streptomycin (50 μg/mL). The cells were adjusted to a density of 1 × 10^5^ cells/mL and planted in culture dishes. A half of culture medium was changed every 3 days. An aggregate form of NSCs, which were named neurospheres, were observed during this procedure at about day 7. Then, neurospheres were collected, dissociated with Accutase (Sigma, USA) for 5 min, and passaged at a cell density of 1 × 10^5^ cells/mL. Cell counting and viability examination is required at every passage, using trypan-blue exclusion method. The five passages of NSC were used for the in vitro and in vivo experiments.

### Immunocytochemistry Staining

For cell seeding in peptides, 30 μL peptide solution was added to evenly cover the bottom surface of each well of 96 multi-well plates (BD Biosciences), followed by slow addition of 200 μL/well of medium containing NSC at a density of 2 × 10^5^ cells/mL. At 2 weeks after being seeded, cultured cells in the group of NSC seeded in DSP (DSP group), NSC seeded in SP (SP group), and NSC alone (control group) were washed briefly with phosphate-buffered saline (PBS) and then fixed with 4 % paraformaldehyde for 20 min, subsequently incubated with blocking solution (10 % goat serum in PBS containing 0.1 % Triton-X100) for 1 h at room temperature. Neuronal differentiation was determined by immunostaining with anti-neuron specific enolase (NSE) (1:200, Abcam, USA) diluted in blocking solution overnight at 4 °C. Goat-raised secondary antibodies were incubated 1 h at room temperature. Cell nuclei were stained with 4ʹ,6-diamidino-2-phenylindole (DAPI;1:1000; Sigma, USA) in PBS for 5 min. Negative controls for immunostaining were also performed.

### RT-PCR Analysis

NSCs were harvested and total RNA was extracted with TRIzol (Roche). The synthesis of the first-strand complementary DNA (cDNA) was carried out from 1 μg of total RNA using reverse transcriptase (Takala) according to the manufacturer’s instructions. After synthesis, cDNA was used in PCR reaction with gene-specific primers. The sequences of the PCR primer pairs (5′ to 3′) that were used for each gene are as follows: nestin, GGAGTGTCGCTTAGAGGTGC (forward) and CAGCAGAGTCCTGTATGTAGCC (reverse); β3-tubulin (Tuj1), TGCGTGTGTACAGGTGAATGC (forward) and AGGCTGCATAGTCATTTCCAAG (reverse); NSE, GTGGACCACATCAACAGCAC (forward) and TGAGCAATGTGGCGATAGAG (reverse); and glial fibrillary acidic protein (GFAP), CTCAATGCTGGCTTCAAGGAGA (forward) and GACGCAGCGTCTGTGAGGTC (reverse). In addition, amplification of β-actin from the same amount of cDNA was used as an endogenous control with the following primers: forward CAATGAGCGGTTCCGATG, reverse GCCACAGGATTCCATACCCA. Products were analyzed on agarose gel and visualized by ethidium bromide staining. The relative amount of each transcript was normalized to the level of β-actin.

### MTS Assay

Aβ_1–40_ was dissolved initially in sterile, distilled water and further diluted with 0.1 M PBS without calcium. The solution of Aβ_1–40_ was incubated for 5 days at 37 °C to allow fibril formation. Aliquots were stored at −80 °C until use. For in vitro cytotoxicity of Aβ_1–40_ on NSC, the Cell Titer 96 AQueous One Solution Cell Proliferation Assay (Promega, France) was tested. NSC were plated at 10,000 cells/well in a 96-well plate in the absence and presence of Aβ_1–40_ (Sigma; 1, 5, 10, 20, 40 μM) in a 96-well plate. According to the manufacturer’s recommendations, 20 μL MTS solution was added into each of the wells at day 2 of cell culture. Then, cells were incubated for 2 h at 37 °C in the humidified 5 % CO_2_ atm incubator, and results were obtained at a wavelength of 490 nm. The same volume of medium without cells was used as blank. Results were expressed in optical density (OD).

### Cell Viability in DSP with or Without Aβ Treatment In Vitro

After seeding NSC within the DSP, the cell viability was observed using a fluorescence microscope by acridine orange/ethidium bromide (AO/EB) staining at 7 and 14 days. The fluorescence dye AO/EB was added to the supernatant. Both AO and EB were at the concentration of 100 μg/mL in PBS. The number of different stained cells was determined by counting 300 cells at five random, non-overlapping fields in five different cell samples. Data were expressed as percentage of survived cells in each group. To further test the neuroprotective effect of DSP on NSC, the cells were simultaneously treated with 40 μM Aβ_1–40_. Then, the morphological changes of the cells were observed by dual staining of propidium iodide (PI) and DNA-binding fluorochrome Hoechst 33342. Apoptosis was observed by Hoechst 33342, characterized by chromatin condensation and nuclear fragmentation, while in the event of late apoptosis and necrosis, loss of membrane integrity could be detected by PI.

### Intra-hippocampally Injected Aβ_1–40_ Rat Model and NSC Transplantation

Animals were randomly assigned to seven experimental groups each comprising 15 SD female rats: the groups of normal, control, NSC, SP, NSC + SP, DSP, and NSC + DSP. All of the rats, except in the normal group, were anesthetized by intraperitoneal injection of sodium pentobarbital (50 mg/kg), and placed into a stereotaxic instrument (David Kopf instruments, USA). An incision was made along the midline, and the area surrounding the bregma was cleaned and dried. Aβ_1–40_ solutions (2.5 μg/μL) were administrated by injections with a Hamilton microsyringe fixed to a 26-gauge needle. Two microliters of Aβ_1–40_ were administered over 5 min into the CA1 area of hippocampus at coordinates incisor bar −3.3 mm, 3.8 mm posterior to the bregma, ±3.2 mm lateral to the sagittal suture, and 2.7 mm down from top of the skull bilaterally [29]. The needle remained in position for an additional 5 min after injection to allow diffusion into the surrounding tissue. After that, transplantation of peptides, or NSC at passage 5 (5 × 10^5^, 5 μL, 1 μL/min) derived from male rats with or without peptides was performed with a new microsyringe. Injection coordinates for the hippocampus were the same as the Aβ injection. In the NSC group, NSC was suspended in 5 μL of PBS and the cell suspension was injected. In peptides alone and control groups, SP or DSP was dissolved in sterile sucrose (295 mmol/L) at 1 % (*w*/*v*) and sonicated for 20 min, 5 μL of SP or DSP and PBS was injected, respectively. In the group of NSC in peptides, NSC were suspended in 5 μL of peptides solution and then injected. After injection, the syringe was left in place for an additional 5 min before it was slowly withdrawn. The incision was closed after and the animal was allowed to recover under a heat lamp. Chloramphenicol (1 % solution) was applied to the exposed skull and scalp before closure to limit local infection.

### Morris Water Maze Test

Four weeks after the transplantation, the training of all experimental groups of animals started in the Morris water maze. This maze included a black-painted circular water tank (150-cm diameter, 60-cm height), filled to a depth of 40 cm with water (22 ± 2 °C). This pool was divided to four equal-spaced quadrants and a hidden transparent platform (10 cm in diameter), made of Plexiglas was located 1 cm below the surface of the water in the center of the north-west quadrant (target quadrant). The task was conducted twice a day for 5 consecutive days. In each trial, the rat was placed in the water at one of four starting positions, with the sequence of the positions being selected randomly. Animals were allowed to swim in the pool during a period of 70 s and find the hidden platform. If an animal did not find the platform within this period, it was manually guided to the platform by the researcher. The rats rested 30 s between two consecutive trials and all trials at about the same time of the morning were performed. Directions of the rats were recorded by a video camera located just above the center of the maze. The camera was linked to a computer. Learning capabilities were tested by measuring escape latency (time to find the platform), and swimming speed parameters. For measurement of post-training probe trial tests, animals were trained for 5 days as described above, and on the next day, post-training probe trial tests were performed. In the probe trial test, the hidden platform was removed, and animals were placed in the pool in the opposite of the target quadrant and allowed to swim freely for 70 s. The occupancy and crossing of animals in proximity of the target quadrant (the quadrant included the hidden platform during training trials) were assessed.

### Fluorescence In Situ Hybridization and Immunohistochemistry Staining

After being fixed with 4 % paraformaldehyde and dehydrated in 30 % sucrose-containing PBS, the tissues were then embedded in OCT (Fisher Scientific) and rapidly frozen in −80 °C. Coronal sections (20 μm) through the hippocampus were cut on a cryostat (Leica CM 3050, Deerfield, IL). For detecting the existence of the transplanted NSC, in situ hybridization using rat Y chromosome specific gene was performed as we described before [30]. To determine the differentiation of transplanted cells, immunohistochemistry for Tuj-1 was performed. The same sections were incubated with mouse monoclonal Tuj-1 antibody (1:200; Abcam) overnight at 4 °C. Synapsin-1 was also detected with rabbit anti-synapsin-1 (1:100, CST). The sections were then incubated with donkey anti-mouse or anti-rabbit antibody (Jackson, USA) for 2 h at room temperature. After washing, the nuclei were counterstained with DAPI. The neuronal apoptosis was estimated by the staining of Hoechst 33342 and the number of apoptotic cells was counted under ×200 fields.

### Western Blot Analysis

Hippocampus tissue from each group was homogenized in ice-cold radio-immunoprecipitation assay (RIPA) lysis buffer containing protease inhibitor phenylmethylsulfonylfluoride (PMSF) with a glass homogenizer on ice. The lysates were centrifuged at 12,000 rpm for 5 min. The supernatant was collected and protein concentration was measured using a BCA protein assay kit (Pierce, Rockford, IL). After boiling for 5 min, an equal amount of total protein (30 or 50 μg each) was separated on 10 or 15 % sodium dodecyl sulfate polyacrylamide gel electrophoresis (SDS-PAGE) and then transferred onto a polyvinylidene fluoride (PVDF) membrane (Millipore, USA). Membranes were blocked for 1 h at room temperature with 5 % (*w*/*v*) nonfat dried milk in Tris-buffered saline Tween-20 (TBST), and incubated with specific primary antibodies against phosphorylated Akt (p-Akt; Ser473; 1:500), Akt (1:1000), caspase-3 (1:2000), cleaved caspase-3 (1:1000), Bcl-2 (1:500), Bax (1:500), and synapsin-1 (1:1000; all from Cell Signaling Technology) overnight at 4 °C. Antibody binding was revealed by incubation with horseradish peroxidase-conjugated anti-mouse or anti-rabbit antibodies (1:5000–10,000; Jackson, USA). Enhanced chemiluminescence (ECL) was detected by exposure to x-ray film, with glyceraldehyde-3-phosphate dehydrogenase (GAPDH) (Cell Signaling Technology) used as an internal control. Densitometric analysis of the bands was performed with an image analyzer (Quantity One v4.62, Bio-Rad).

### ELISA

To evaluate the presence and degradation of the injected Aβ, hippocampus and cortex tissues were homogenized in 10 volumes of lysis buffer with protease inhibitor mixture (Complete, Mini, EDTA-free, Roche). After centrifugation at 10,000×*g* for 15 min at 4 °C, supernatants were assessed using the human Aβ_1–40_ enzyme-linked immunosorbent assay (ELISA) kit (Immuno-Biological Laboratories, Japan). In brief, 100 μL of sample was added into the precoated 96-well assay plate and incubated overnight at 4 °C. Then, the wells were washed three times and 100 μL of labeled antibody solution was added to each well for 1 h at 4 °C in the dark. After washing with three times, 100 μL of chromogen solution was added. The samples were gently mixed and incubated for 15 min at 37 °C in the dark. Then, 50 μL of stop solution was added into each well and the absorbance was examined at 450 nm with a microplate reader (Bio-Rad, USA) within 15 min. The expression of BDNF, ciliary neurotrophic factor (CNTF), insulin-like growth factor 1 (IGF-1), TNF-α, IL-1β, and IL-10 was also detected through the specific ELISA kit.

### Quantitative Real-Time PCR

Four weeks after transplantation, rats were killed and hippocampal tissues were extracted and homogenized on ice immediately. Subsequently, total RNA was extracted from each sample TRIzol reagent according to the manufacturer’s procedures, and the concentration was assessed using a NanoDrop ND-1000 spectrophotometer (Thermo Scientific, USA). A total of 1 μg of each template RNA was converted to the first strand of cDNA. The survival of transplanted NSC was determined by the expression of SRY through PCR as previously described. Real-time PCR of cDNA was performed (ABI PRISM 7500 Sequence Detection System, Applied Biosystems) using SYBR Green ready mix (Applied Biosystems) and the forward and reverse primer. The primers for each gene were: BDNF, forward TCTACGAGACCAAGTGTAATCC, reverse TATGAACCGCCAGCCAAT; ciliary neurotrophic factor (CNTF), forward AAACACCTCTGACCCTTCAC, reverse AGTCATCTCACTCCAACGAT; insulin-like growth factor 1 (IGF-1), forward CTGGCACTCTGCTTGCTCAC, reverse CTCATCCACAATGCCCGTCT; TNF-α, forward GCCCACGTCGTAGCAA, reverse GTCTTTGAGATCCATGCCAT; IL-1β, forward GAGCTGAAAGCTCTCCACCT, reverse TTCCATCTTCTTCTTTGGGT; IL-10, forward CAGAAATCAAGGAGCATTTG, reverse CTGCTCCACTGCCTTGCTTT; and glyceraldehyde-3-phosphate dehydrogenase (GAPDH), forward GGAAAGCTGTGGCGTGAT, reverse AAGGTGGAAGAATGGGAGTT. GAPDH was used as an endogenous control to normalize expression levels. Real-time PCR data was analyzed using comparative critical threshold (Ct), and the relative expression levels were calculated according to the formula: 2^−ΔΔCt^ [31]. The specificity was verified by melt curve analysis and agarose gel electrophoresis.

### Statistical Analysis

Data are expressed as the means ± standard deviation. To analyze the data statistically, we performed one-way analysis of variance (ANOVA) with S-N-K post hoc multiple comparison analysis. A value of *P* < 0.05 was considered to be statistically significant.

## Results

### Structures of Designer Self-Assembling Peptide

**Fig. 1:**
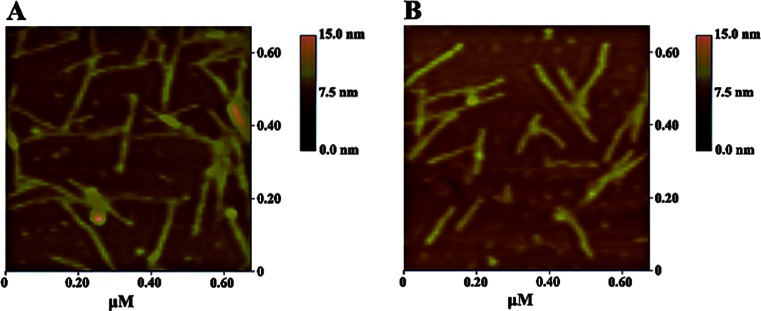
Tapping mode AFM images of peptides RADA16 (**a**) and RADA16 modified with YIGSR (**b**). The appended functional motif did not prevent peptide self-assembly

There was concern that the incorporation of appended motif to the RADA16 peptide would prevent peptide self-assembly. Thus, we used AFM to examine the nanofiber structures of the designer peptide to address this concern. AFM images demonstrated that RADA16 can undergo spontaneous assembly into well-ordered nanofibers in the presence of solutions that contain monovalent salts. Meanwhile, nanofibers ~10 nm in fiber diameter could be fabricated by the spontaneous self-assembly of the designer peptide introduced to physiological salt-containing solutions (Fig. 1a, b). It was evident that the appended functional motif did not inhibit the self-assembling peptide nanofiber formation.

### Designer Self-Assemble Peptide was Beneficial to the Differentiation of NSC

**Fig. 2:**
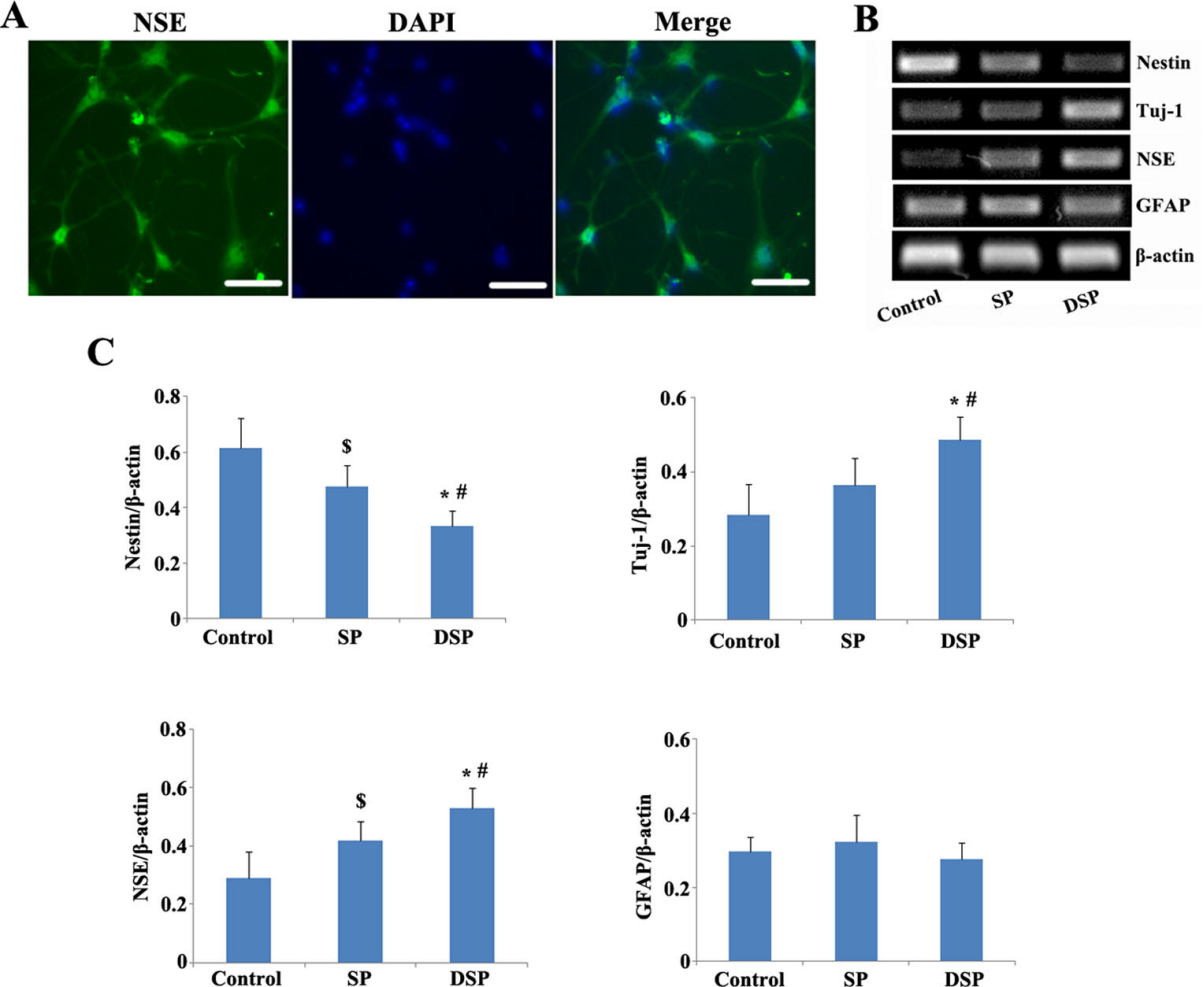
Designer self-assemble peptide was conducive to the differentiation of NSC in vitro. **a** The differentiation of NSC in DSP toward to neuron was detected by NSE immunocytochemistry staining. *Scale bar* 100 μm. **b** PR-PCR showed the expression of different genes 2 weeks after being seeded in each group. **c** Quantitative analysis of the genes expression. *P < 0.01 and ^$^ *P* < 0.05 versus control; ^#^ *P* < 0.05 versus SP

The differentiation of NSC toward neuron was detected through immunocytochemistry and RT-PCR to estimate the effect of functional motif-modified self-assembling peptide in vitro. According to the results of immunocytochemistry, most of the cells in DSP apparently expressed NSE, one of the glycolytic enzymes distributed exclusively in neurons (Fig. 2a). RT-PCR demonstrated that the expression of nestin, a widely employed marker of multipotent NSC, was much lower in the DSP group at 2 weeks after being seeded than that in the SP or control group, indicating that much more NSC has differentiated to others cells. Interestingly, the cells seeded in DSP expressed more Tuj-1 and NSE than other groups, which demonstrated that neuronal differentiation was facilitated through YIGSR modification of RADA16. Compared with the SP and control groups, less cells in the DSP group expressed the astrocyte marker GFAP, although there were no significant differences among the three groups (Fig. 2b, c).

### DSP Promotes Cell Survival Under Normal Condition and Aβ Treatment

**Fig. 3:**
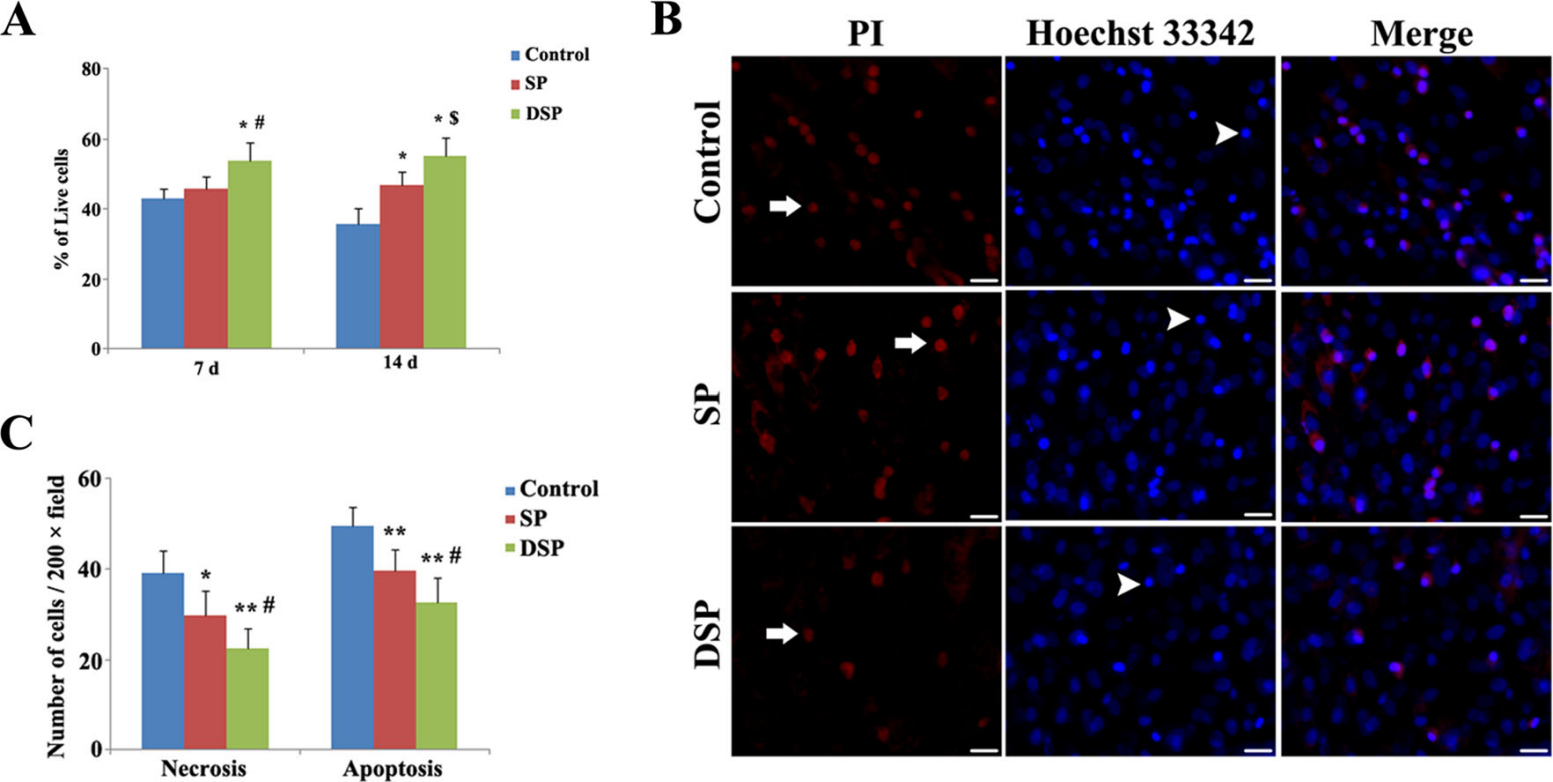
Designer self-assemble peptide increased the cell viability and protected NSC to against the cytotoxicity of aggregated Aβ. **a** Cell viability was detected by AO/EB staining at 7 and 14 days after being seeded in DSP. NSC in modified peptides showed increased cell survival compared to the unmodified RADA16. *P < 0.01 versus control; ^#^ *P* < 0.01 and ^$^ *P* < 0.05 versus SP. **b**, **c** Cell survival after Aβ_1–40_ treatment. PI/Hoechst 33342 staining demonstrated that DSP decreased the number of apoptotic NSC compared to the unmodified SP, suggesting more efficient to against the neurotoxic of Aβ. *White arrow* indicated PI-positive cells and *arrowhead* indicated Hoechst 33342-positive cells. *Scale bar* 20 μm. *P < 0.05 and **P < 0.01 versus control; ^#^ *P* < 0.05 versus SP

The AO/EB double staining was performed to analyses cell survival in DSP at 7 and 14 days after being seeded. At both time points, the highest percentage of living cells was observed in the functionalized peptide containing the YIGSR functional motif, which suggests that this amino acid sequence may be important to support NSC viability (Fig. 3a). The MTS assay was used to determine Aβ_1–40_-induced toxicity (Fig. S1). A 24-h exposure to Aβ_1–40_ induced a toxic, dose-dependent effect on NSC, with a maximal effect of ~60 % noted at a concentration of 40 μM. Thus, 40 μM was used as the concentration for Aβ_1–40_ to test the neuroprotective effect of DSP on NSC. After treatment with Aβ, most of NSC displayed apparent morphological changes of necrosis and apoptosis in the control group by PI/Hoechst 33342 double staining. Although in the presence of SP, there were many necrotic and apoptotic cells observed. However, the necrosis and apoptosis of NSC was obviously suppressed in the DSP group (Fig. 3b). The number of necrotic and apoptotic cells were both significantly decreased compared with that in the control and SP groups (Fig. 3c).

### NSC Transplantation in Designer SP Rescued Behavioral Impairment

**Fig. 4:**
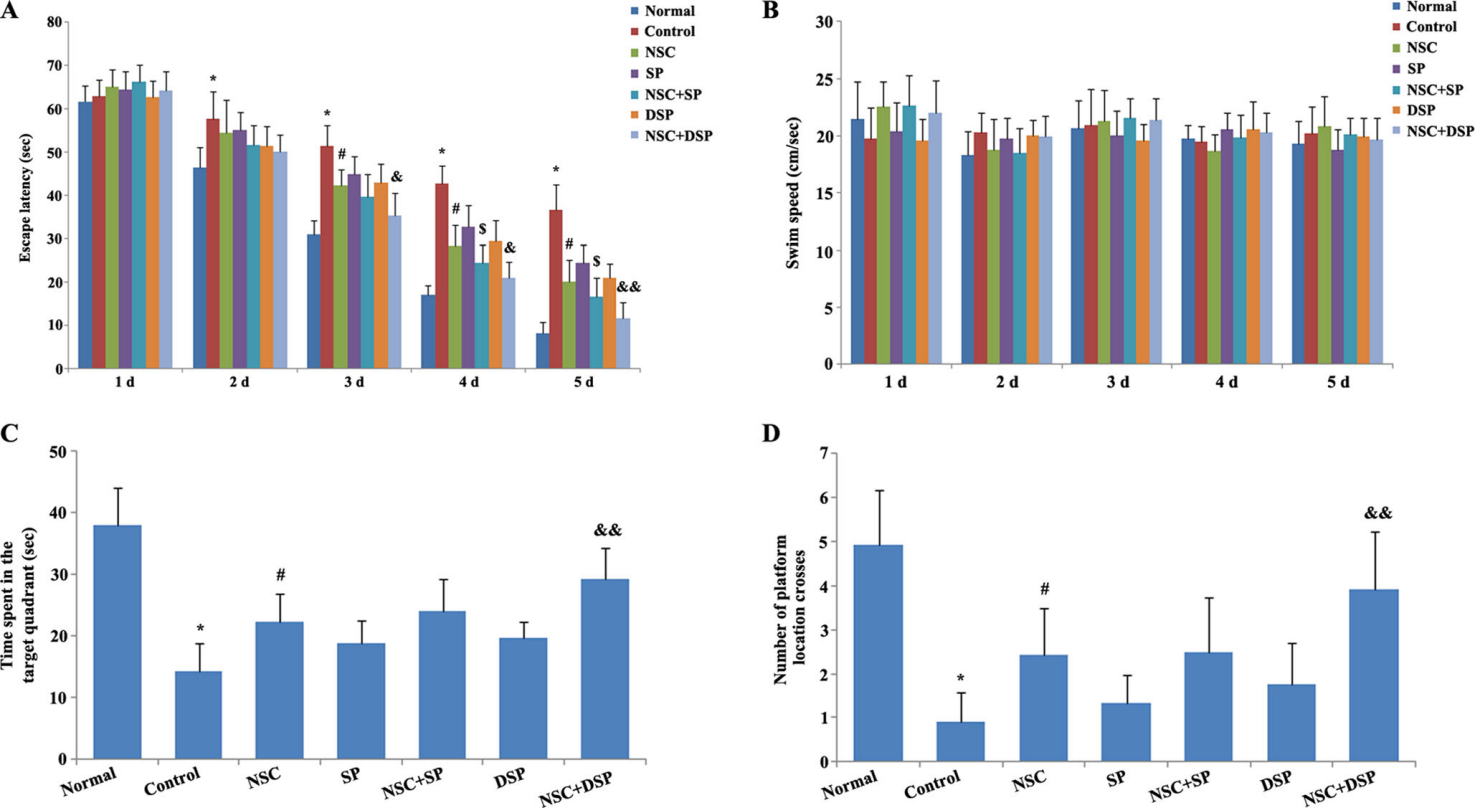
Inhibitory effects of NSC transplantation in DSP on memory impairment in Aβ_1–40_-infused rats model. **a** NSC transplantation in DSP improved spatial learning in AD rats. From day 3, there was a remarkable difference between the group of NSC transplantation in DSP and NSC transplantation in SP. Spatial learning was measured as escape latencies per day. **b** Swimming speed was not significantly different between groups. **c**, **d** NSC transplantation in DSP-restored spatial memory in the AD rats. Rats spatial memory was evaluated by the number of time spent in the target quadrant and platform location crosses. There was no apparent difference was observed between NSC and NSC in SP group. However, rats received NSC transplantation in DSP remained in the original platform quadrant longer and crossed the former quadrant containing the platform more frequently compared to NSC carried in SP-treated rats. *P < 0.01 versus normal, ^#^ *P* < 0.01 versus control, ^$^ *P* < 0.05 versus NSC, ^&^ *P* < 0.05, and ^&&^ *P* < 0.01 versus NSC + SP

The effects of NSC transplantation in DSP on learning and memory ability in rats exposed to Aβ_1–40_ were assessed using the Morris water maze test described above. Spatial learning was measured as escape latencies per day. In the place navigation trials, compared with normal group, the escape latency was obviously increased (days 2~5) in Aβ_1–40_ treated rats. Rats in all of the treat groups displayed significantly shorter latencies than those of the model rats on days 3~6 of training, indicating improvements in spatial acquisition. Compared with the NSC group, NSC transplantation in SP could decrease the escape latency significantly in days 4 and 5. However, rats in the group of NSC transplantation in DSP had the shortest latency among the treated groups. From day 3, there was a remarkable difference between the group of NSC transplantation in DSP and NSC transplantation in SP (Fig. 4a). Swimming speed was not significantly different between groups during the 5 days (Fig. 4b). In the spatial probe trials, NSC transplantation effectively increased the time spent in the target quadrant and number of platform location crosses, indicating impaired spatial memory recall. Although SP could facilitate the efficiency of NSC transplantation, no apparent difference was observed between NSC and NSC in SP group. However, rats received NSC transplantation in DSP remained in the original platform quadrant longer and crossed the former quadrant containing the platform more frequently compared to NSC carried in SP-treated rats, suggesting NSC injection in DSP exerted much more efficient improvement in behavior recovery (Fig. 4c, d).

### DSP Promoted the Survival and Neuronal Differentiation of Transplanted NSC

**Fig. 5:**
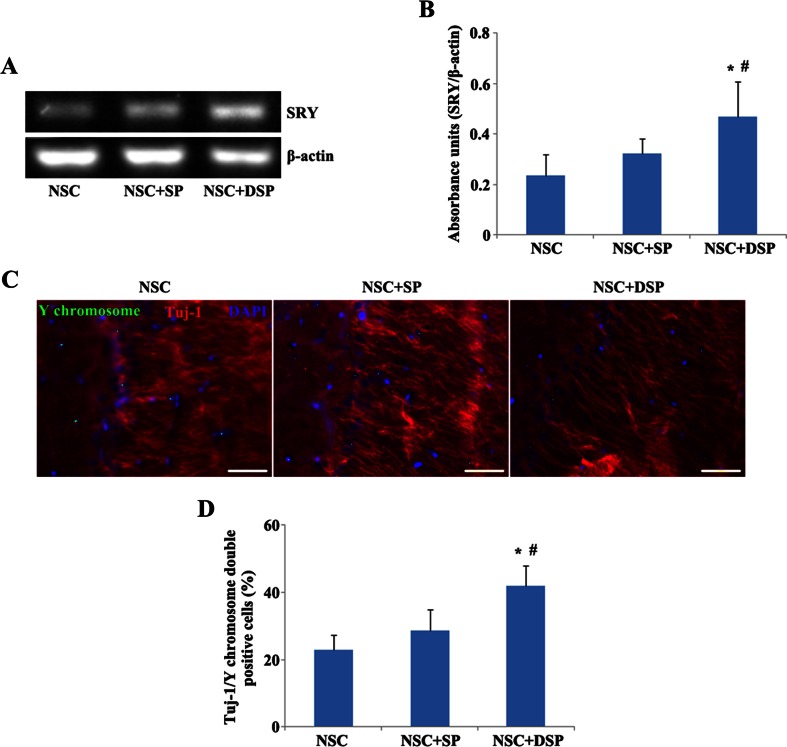
DSP promoted the survival and neuronal differentiation of transplanted NSC. **a** RT-PCR for the SRY gene using hippocampus tissue from NSC transplantation groups with or without peptides. **b** Quantitative analysis of SRY gene expression. *P < 0.01 versus NSC, ^#^ *P* < 0.05 versus NSC + SP. **c** Expression of Tuj-1 by Y chromosome-positive cells. Much more Y chromosome-positive cells in CA1 regions co-expressed Tuj-1 than that in NSC and NSC + SP groups. *Scale bar* 50 μm. **d** Quantitative analysis of the percentage of Tuj-1 and Y chromosome double positive cells in all of the Y chromosome-positive cells. *P < 0.01 versus NSC, and ^#^ *P* < 0.01 versus NSC + SP

The survival of transplanted NSC derived from male rats was monitored by using the SRY gene, which triggers embryonic development as a male in mammals. RT-PCR for the SRY gene using hippocampus tissue from NSC transplantation groups with peptides demonstrated that both peptides could be conducive to the expression of SRY (Fig. 5a). Expression of SRY in the group of NSC in SP was higher than that in the NSC group. Moreover, NSC transplantation in DSP showed much more SRY genes compared with the group of NSC in SP, which indicated that NSC transplanted within DSP represented the highest survival rate (Fig. 5b). Expressions of Tuj-1 and Y chromosome in CA1 region were detected by immunohistochemical staining to estimate the differentiation of NSC toward neuron in vivo (Fig. 5c). There were much more Y chromosome-positive cells in the group of NSC in DSP than that in the group of NSC in SP, which also demonstrated the best survival rate in the group of NSC in DSP. SP could contribute to the expression of Tuj-1 in Y chromosome-positive cells. Interestingly, a significantly higher fraction of Y chromosome-positive cells were labeled by Tuj-1 in the group of NSC in DSP than that in the group of NSC in SP, suggesting that neuronal differentiation of transplanted NSC may be facilitated through the addition of functional motif to SP (Fig. 5d).

### NSC Transplantation in DSP Prevented the Apoptosis Induced by Aβ

**Fig. 6:**
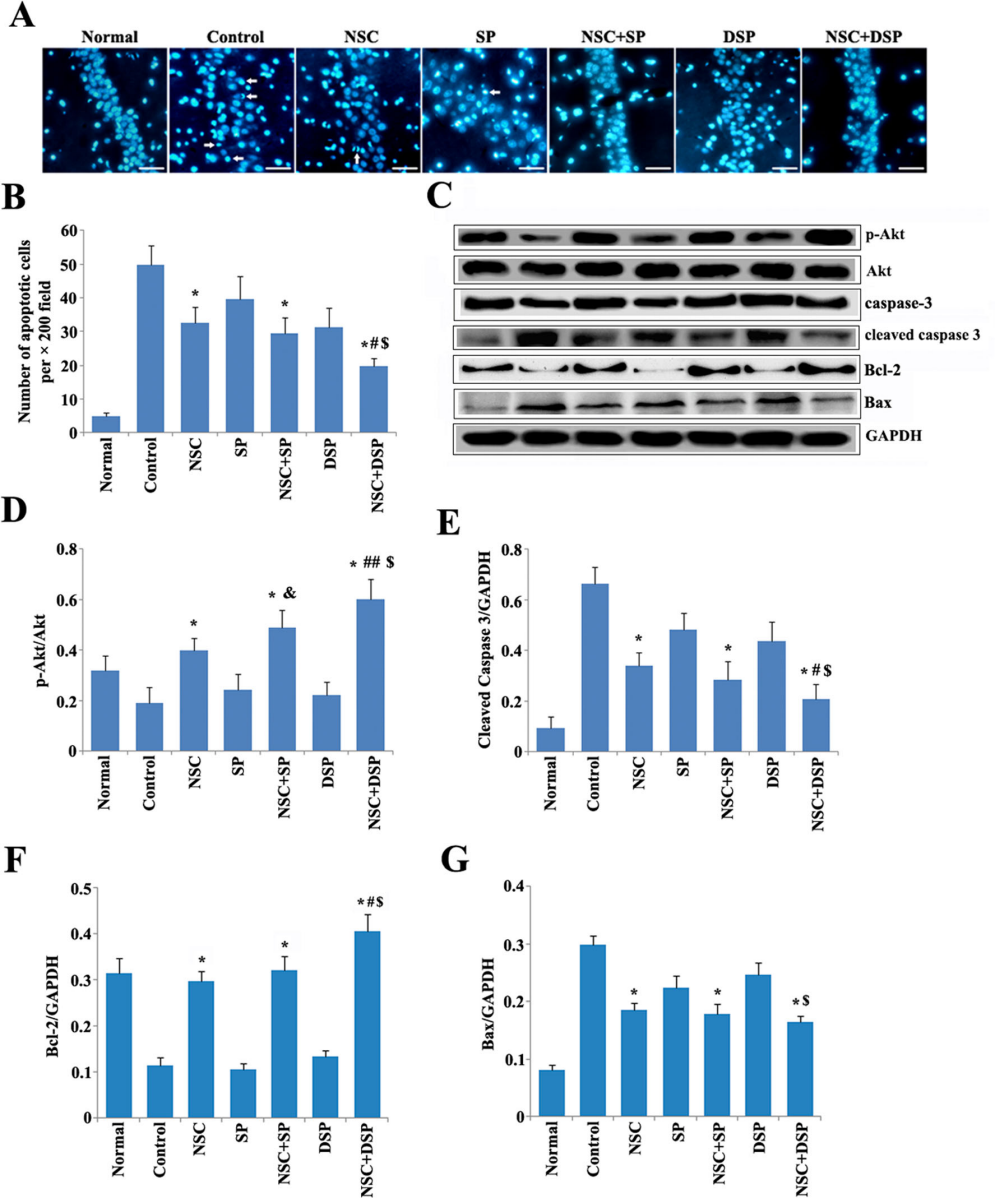
NSC transplantation in DSP prevented the apoptosis induced by Aβ in hippocampus tissue through upregulation of p-Akt, downregulation of cleaved caspase 3 and balancing the ratio of Bcl-2 to Bax. **a** Apoptosis was detected through Hoechst 33342 staining in CA1 region. *White arrow* indicated Hoechst 33342-positive cells. *Scale bar* 30 μm. **b** Quantitative analysis of Hoechst 33342-positive cells. *P < 0.01 versus control, ^#^ *P* < 0.01 versus NSC + SP, ^$^ *P* < 0.01 versus DSP. **c** Expression of p-Akt, cleaved caspase 3, Bcl-2 and Bax in hippocampus tissue examined by Western blot. **d**–**g** Quantitative analysis of the expression of p-Akt, cleaved caspase 3, Bcl-2, and Bax. *P < 0.01 versus control, ^#^ *P* < 0.05 and ^##^ *P* < 0.01 versus NSC + SP, ^$^ *P* < 0.01 versus DSP, and ^&^ *P* < 0.05 versus NSC

We explored whether NSC transplantation in DSP was able to improve memory impairment by preventing Aβ_1–40_ induced neuronal cell death in the hippocampus. Apoptosis was detected through Hoechst 33342 staining. The apoptotic nucleus appears smaller, condensed, and deeply stained. The results showed that there were few apoptotic neurons in the normal group, while in the control group, the apoptotic cells significantly increased. Compared with the control group, all of the treatments significantly alleviated the apoptotic cells in the hippocampus CA1 region, especially in the groups of NSC in SP and NSC in DSP (Fig. 6a). Although SP decreased the number of apoptotic cells, the difference of neuronal apoptosis between the groups of NSC and NSC in SP was indistinguishable. The number of apoptotic cells in the group of NSC in DSP was much less than that in the groups of NSC in SP and DSP (Fig. 6b). Moreover, the expression of cleaved caspase 3 and p-Akt, known to be involved in the process of apoptosis, were examined by Western blot in hippocampus tissue. The control group represented the highest cleaved caspase 3 and lowest p-Akt level. NSC transplantation alone or in SP could downregulate the expression of cleaved caspase 3 and upregulate the expression of p-Akt. Expression of p-Akt appeared with significant difference between the group of NSC and NSC in SP, while no statistic difference in the expression of cleaved caspase-3. However, cleaved caspase 3 was lower and p-Akt was higher in the group of NSC in DSP than that in the group of NSC in SP. There was no significant difference in caspase-3 expression among the groups. The expression of the proteins involved on caspase regulation was further detected. NSC transplantation increased the level of Bcl-2 and decreased the level of Bax. Samples from the group of NSC + DSP exhibited much higher level of Bcl-2 than the group of NSC + SP. These data indicated that NSC transplantation in DSP prevented the apoptosis induced by Aβ in hippocampus tissue through contributing to the upregulation of p-Akt, downregulation of cleaved caspase 3 and then balancing the ratio of Bcl-2 to Bax (Fig. 6c–g).

### NSC Transplantation in DSP was Beneficial to the Recovery of Synaptic Function

**Fig. 7:**
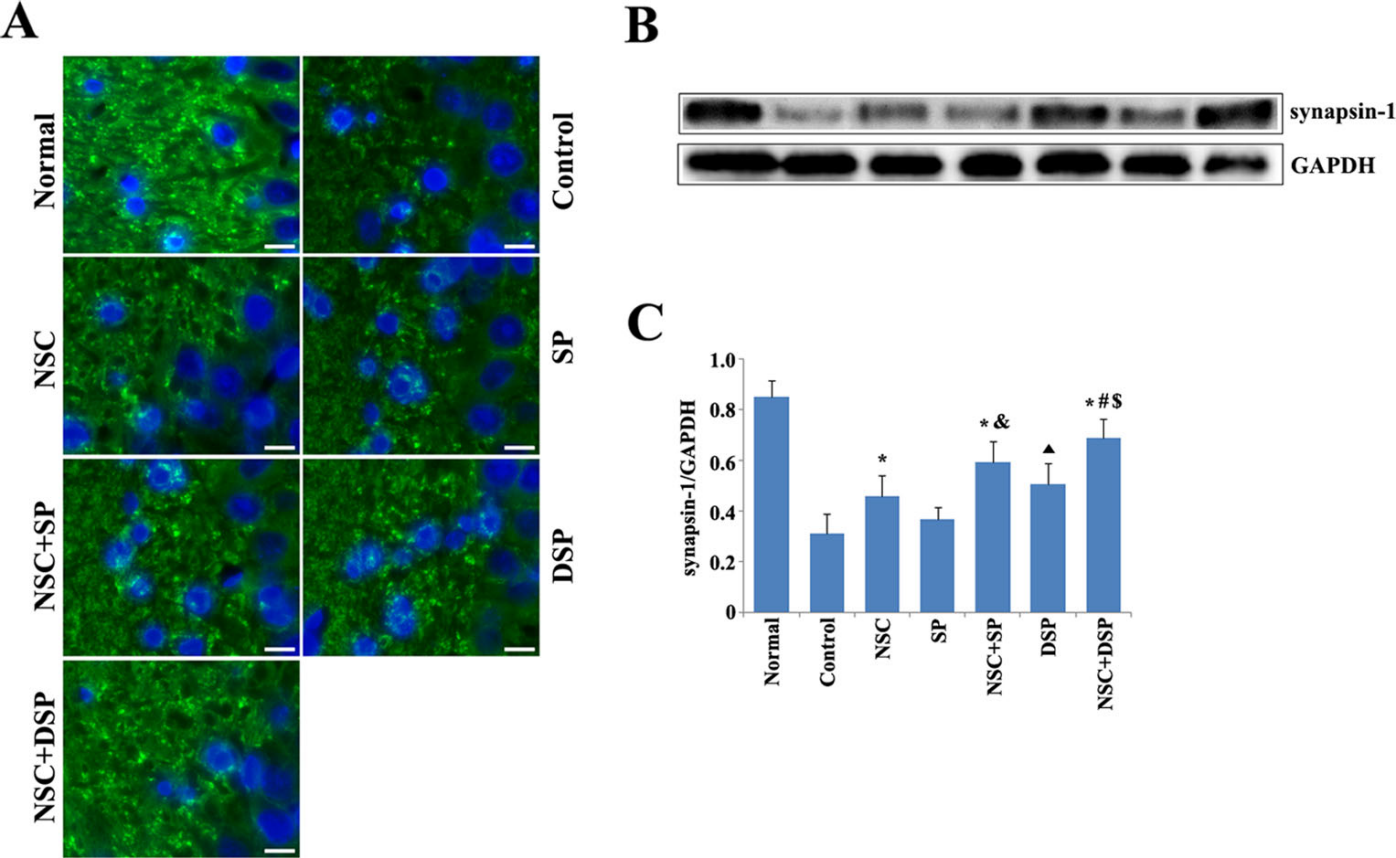
NSC transplantation in DSP was benefit to the recovery of synaptic function. **a** Immunohistochemical analysis of synapsin-1 in CA1 region. *Scale bar* 10 μm. **b** Expression of synapsin-1 was detected by western blot. **c** The signals of synapsin-1 were normalized using GAPDH. *P < 0.01 versus control, ^#^ *P* < 0.05 versus NSC + SP, ^$^ *P* < 0.01 versus DSP, ^&^ *P* < 0.01 versus NSC, *black triangle P* < 0.05 versus SP

Synaptic proteins are important for the normal functioning of synapses. We further investigated the expression of pre-synaptic protein synapsin-1 in hippocampus by immunohistochemistry staining and western blot (Fig. 7a, b). We found that the level of synapsin-1 was markedly reduced in the Aβ-treated rats. The level of synapsin-1 in the hippocampus was markedly elevated in the groups of NSC, NSC in SP, DSP, and NSC in DSP, but not in the SP group. Interestingly, DSP injection alone was beneficial to the restoration of synaptic function, and represented more advantage than SP transplantation. There was no significant difference between the groups of NSC alone and DSP alone. More importantly, NSC transplantation in DSP increased the expression of synapsin-1 in the most extent compared with other groups (Fig. 7c).

### Inhibitory Effect of NSC Transplantation in DSP on Aβ_1–40_

**Fig. 8:**
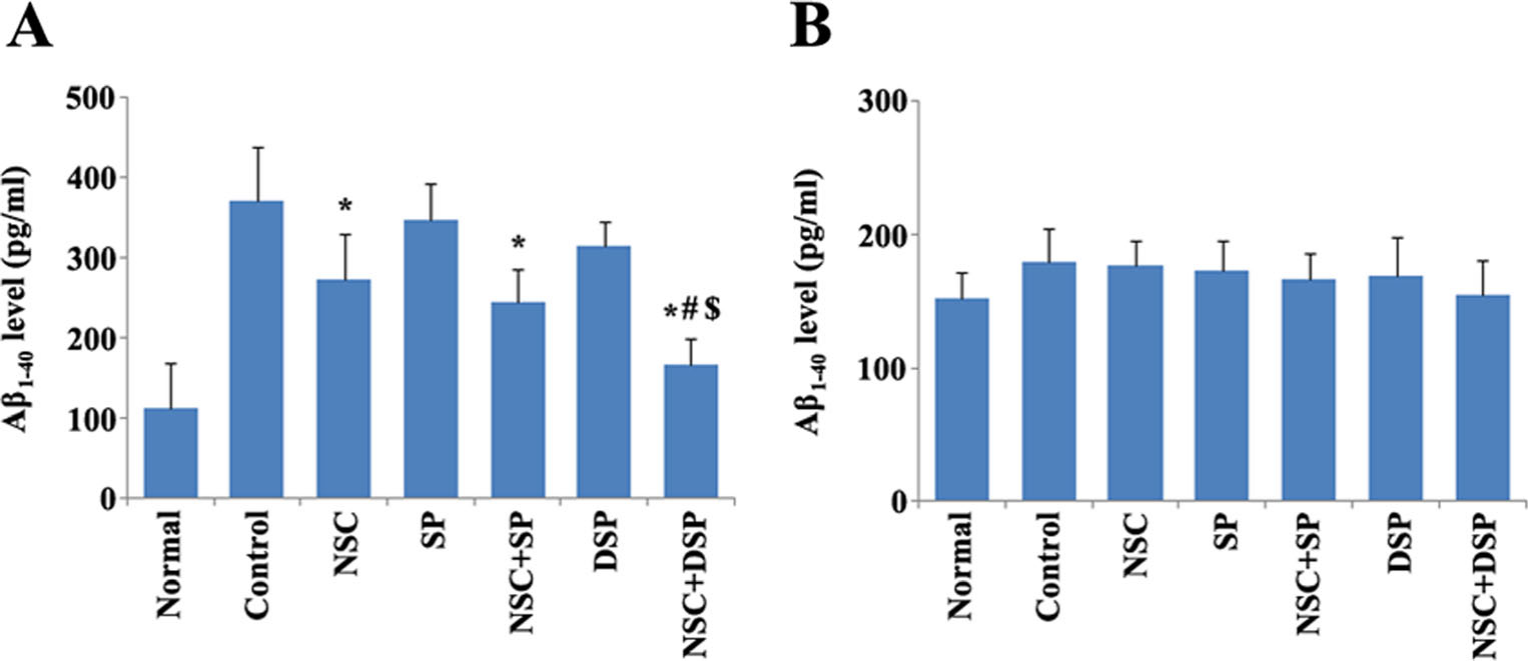
Inhibitory effect of NSC transplantation in DSP on Aβ_1–40_ in the brain of AD rats. Aβ_1–40_ level in rat hippocampus tissue (**a**) and cortex (**b**) was measured by ELISA. *P < 0.01 versus control, ^#^ *P* < 0.05 versus NSC + SP, ^$^ *P* < 0.01 versus DSP

Aβ_1–40_ level in rat hippocampus tissue and cortex was measured by ELISA to examine the inhibitory effect of NSC transplantation in DSP on Aβ_1–40_. As shown in Fig. 8a, although Aβ could be degraded in some extent by the endogenous enzymes, Aβ_1–40_ level in hippocampus tissue was very high in the control group. NSC transplantation with or without peptides obviously attenuated the Aβ_1–40_ level in hippocampus tissue. The expression of Aβ_1–40_ in the group of NSC in SP was similar to the NSC group, indicating that SP couldn’t play a potential synergistic effect with NSC on downregulation of Aβ in hippocampus tissue. NSC transplantation in DSP showed the lowest Aβ_1–40_ level among all of the treated groups, and there were significant differences between the groups of NSC in DSP and DSP alone or NSC in SP. However, Aβ_1–40_ level in cortex was no significant differences among all of the groups including normal and control groups (Fig. 8b).

### Determination of Neurotrophin and Inflammatory Cytokines RNA Levels

**Fig. 9:**
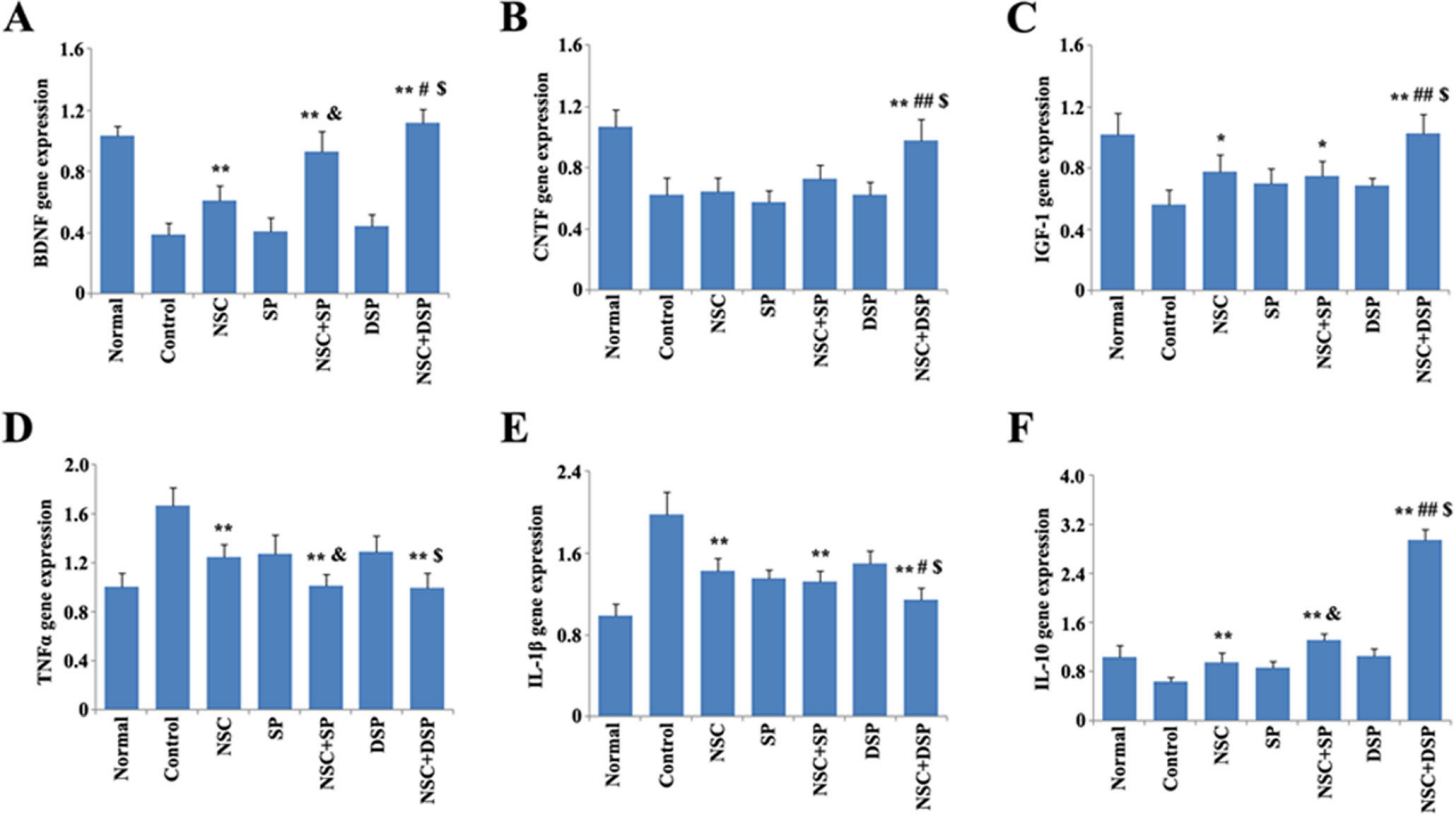
Determination of neurotrophin and inflammatory cytokine RNA levels after NSC transplantation. Quantitative real-time PCR of mRNA expression of neurotrophin (**a**–**c**) and inflammatory cytokines (**d**–**f**) in each group at 4 weeks after transplantation. *P < 0.05 and **P < 0.01 versus control, ^#^ *P* < 0.05 and ^##^ *P* < 0.01 versus NSC + SP, ^$^ *P* < 0.01 versus DSP, ^&^ *P* < 0.05 and ^&&^ *P* < 0.01 versus NSC

The messenger RNA (mRNA) expression of neurotrophin and inflammatory cytokines in each group at 4 weeks after transplantation was detected by quantitative real-time PCR. The expression of neurotrophin cytokines was significantly increased after NSC transplantation except CNTF. NSC transplantation with SP significantly promoted the expression of BDNF compared with NSC treatment alone. Expression of all of the neurotrophin in the group of NSC in DSP, including BDNF, CNTF, and IGF-1, was much higher than that in DSP group and NSC + SP group (Fig. 9a–c). Aβ injection could obviously trigger the upregulation of pro-inflammatory cytokines TNF-α and IL-1β, whereas the downregulation of anti-inflammatory cytokine IL-10. NSC transplantation could attenuate this phenomenon with significant difference. Samples from NSC transplantation in SP had a lower level of TNF-α and higher level of IL-10 than that from NSC transplantation alone. There was no statistically significant difference in IL-1β between the group of NSC and NSC in SP. Compared with the group of NSC transplantation with SP, NSC transplantation with DSP developed a much higher expression of IL-10 and lower expression of IL-1β. However, no significant difference was observed in the expression of TNF-α between the group of NSC + SP and NSC + DSP (Fig. 9d–f). NSC transplantation in DSP also increased the protein level of BDNF, CNTF, and IL-10 compared to the groups of DSP and NSC + SP, while it decreased the expression of IL-1β (Supplementary Table 1).

## Discussion

Stem cells with the potential to form many different cell types are being actively investigated for their potential to replace damaged or dead cells after injury [32]. However, the efficacy of the procedure remains hampered by a low rate of sustained cell engraftment and differentiation toward neurons [16]. In order to enhance the therapeutic efficacy, many studies have reported benefits from biomaterials to provide a suitable scaffold for transplanted stem cells. Specifically, self-assembling peptides that can form 3D structures of nanofibers without apparent immune response have been developed. These peptides are promising biomaterials for spinal cord repair as they can easily be injected into the lesion site and can provide physical support to regrowing nervous tissue [33]. Recently, lots of bioactive motifs identified from extracellular matrix proteins, have been directly coupled on to the self-assembling peptide to create a more advantageous microenvironment niche to the survival and differentiation of the stem cells. But to date, there was no report about the application of stem cell transplantation with designer self-assembling peptide for the treatment of AD.

In this study, we firstly utilized the designer peptide with bioactive motif YIGSR to facilitate the survival and neuronal differentiation of NSC after being transplanted into the hostile microenvironment in hippocampus tissue. YIGSR, one of the laminin-derived bioactive peptides, had similar effect to laminin on the migration and differentiation of neural stem/precursor cells (NSPCs), thus suggesting biomaterials modified with YIGSR maybe a potential approach to improve migration, survival, and neuronal differentiation of stem cells for neural tissue engineering applications [26]. In our study, a single infusion of Aβ_1–40_ effectively impaired learning and memory behavior, which was consistent with other reports [34, 35]. Although these rat models do not reproduce the full complexity of the human disease, they do display a neurodegenerative process and local microglial activation—both usually absent in transgenic mice models of AD [36]. Compared to Aβ injection rats, rats receiving NSC showed reduced latency in finding the hidden platform, spatial learning acquisition. The data confirmed that NSC transplantation is able to reduce the impairment of spatial memory associated with the accumulation of Aβ peptide. Compared with the NSC group, NSC transplantation in SP could decrease the escape latency significantly after 3 days training. But in the spatial probe trials, no apparent difference was observed between NSC and NSC in SP groups. However, rats in the group of NSC transplantation in DSP had a much shorter latency and remained in the original platform quadrant longer and crossed the former platform contained quadrant more frequently compared to NSC carried in SP-treated rats. All of these data suggested that NSC injection in DSP exerted a much more efficient improvement of behavior function after Aβ injection.

The main goal of this study was to increase the survival and neuronal differentiation of NSC. We first investigated the toxicity effect of Aβ on NSC in vitro, and the data indicated that DSP could not only benefit the viability of NSC in normal culture condition but also suppress effectively the necrosis and apoptosis induced by Aβ. These results provided a direct hint to support the hypothesis that survival of grafted NSC was hampered in vivo. From our study, DSP indeed could increase the survival of NSC after transplanting according to the expression of SRY. It has been found that IKVAV-functionalized self-assembling peptide matrix could favor neuronal differentiation of embryonic stem cells [37]. In our study, there were more survived cells after transplantation with DSP co-expressing the neuron marker Tuj-1 than that in the group of NSC in SP. Neuronal differentiation was facilitated through YIGSR modification of RADA16 was also confirmed in vitro, evidence by NSC showed remarkably neuronal differentiation after seeded in DSP and the cells expressed much more neuron-related genes including Tuj-1 and NSE than that cultured in SP. These data demonstrated that neuronal differentiation of transplanted NSC may be facilitated through the addition of YIGSR to RADA16. On the other hand, the adhesion and interaction between NSC and DSP, which may enhance the secretion of neurotrophic factors, could also partly interpret this benefit. A recent study found that bioactive motif-functionalized self-assembling peptides may constitute promising biomimetic scaffolds for in vitro NSC differentiation, as well as regenerative therapy of the acute contusive spinal cord injury in rats [38]. In AD mouse models, it has been reported that impaired capacity for hippocampal neuron replacement may contribute to the cognitive decline [39]. Therefore, it is valuable to utilize the functional motif-modified self-assembling peptides to optimize the survival and neuronal differentiation of NSC according to our and other studies. However, we didn’t investigate the function of the neurons differentiated from NSC, although DSP was facilitated to the neuronal differentiation. To test whether neurons differentiated from NSC have functional activity, sodium currents, potassium currents, action potentials, and synaptic events could be recorded and analysed with electrophysiological technique. The function of the neurons differentiated from NSC was worth to be explored and clarified in the future research.

Neuronal loss and synaptic degeneration is the ultimate event and the main cause of irreversible progression of neurodegenerative disorders [34, 40]. Reducing the level of apoptosis of hippocampal neurons in acute AD would be the key strategy to restore the learning and remembering function. It is well established that the oligomeric forms of the peptide are neurotoxic as well as synapse toxic [41]. In the present study, we found that after injection of Aβ, many cells in CA1 regions demonstrated obvious apoptotic morphology. NSC transplantation with or without peptides could decrease the apoptosis in CA1 regions. The number of apoptotic cells in the group of NSC in DSP was much less than that in the groups of NSC and NSC in SP. Previous studies demonstrated that caspase 3 activation is one of the main pathways involved in Aβ-induced cell death and caspase inhibitors could prevent Aβ-induced apoptosis in rats [42]. The PI3K/Akt signaling pathway is a classic anti-apoptosis signal transduction pathway in the cells. We found that NSC transplantation in DSP prevented the neuronal apoptosis induced by Aβ in hippocampus tissue through the PI3K/Akt signaling pathway in a caspase-3-dependent manner.

Reduction in the number of synapses has been reported in normal aging human subjects and AD patients [43]. Synaptic proteins are essential components to maintain normal synaptic function. Synapsin-1 is one of the pre-synaptic proteins which regulate neurotransmitter release. Our data showed that NSC transplantation could alleviate the downregulation of synapsin-1 induced by Aβ injection. Although SP was beneficial to the recovery of synapsin-1 expression when injected along with NSC, NSC transplantation in DSP showed more preferential expression of synapsin-1 than that in the group of NSC in SP. More interestingly, the YIGSR-functionalized peptide DSP exerted much more benefit than SP injection alone, which was worthy to be investigated further. Synapsin-1 could control the fraction of synaptic vesicles available for release through changing its state of phosphorylation [44]. However, other proteins related to the synaptic function, such as synaptophysin and PSD95, need to be further examined.

Accumulation of deposits of Aβ is thought to be responsible for neuronal and synaptic loss, leading to progressive cognitive deficits seen in AD. Aβ can interact with various cellular components to trigger signal transduction cascades that prompt caspase activation, inflammatory response, free-radical generation, and Ca^2+^ deregulation [45]. Each of these factors can act independently or in concert to damage neurons and disturb cognitive processes. We found that the treatment of NSC transplantation in peptides, but not NSC alone, decreased the levels of Aβ_1–40_ in the hippocampus as demonstrated by ELISA. More important, the expression of Aβ_1–40_ in the hippocampus in the group of NSC in DSP was much lower than that in the group of NSC in SP. The downregulation of Aβ_1–40_ by NSC transplantation in DSP was not only helpful to suppress the progress of progression of neurodegeneration, reduce the apoptosis and synaptic loss, but also contribute to the survival of NSC.

To better understand the effect of DSP on NSC transplantation, we moved forward to test the expression of inflammatory cytokines and neurotrophic factors to determine whether the interaction of DSP and NSC enhanced the paracrine action. TNF-α and IL-1β as pro-inflammatory cytokines, have been related to the cognitive decline characteristic of AD and proposed to be valuable biomarkers for this disorder [46]. Reports demonstrate that inflammatory responses mediated by Aβ peptides in the brains of AD were significantly elevated, such as IL-1β and TNF-α, which is believed to mediate neuronal injury and finally, cognitive decline [47]. IL-10 is a pleiotropic cytokine and inhibits the synthesis and release of pro-inflammatory cytokines such as TNF-α and IL-1β, -6, -8, and -12. We found that NSC transplantation in DSP significantly decreased the expression of IL-1β and increased the expression of IL-10 in hippocampus compared with NSC transplantation in SP. IL-10 could suppresses caspase-3-mediated neuronal apoptosis [48]. Moreover, virus-mediated expression of IL-10 in laterally hemisectioned spinal cords promotes neuronal survival and improves motor function, both of which are associated with activation of glycogen synthase kinase 3-β, Akt, and STAT3 [49]. Neurotrophic factors, such as BDNF, CNTF, and IGF-1, play important regulatory roles in the development, survival, and maintenance of specific neuronal populations [10, 11]. Neurological improvement may partly be due to the increase of neurotrophic factors in hippocampus tissue after NSC transplantation. NSC elevated hippocampal BDNF, leading to increased synaptic density and restoring hippocampal-dependent cognition [13]. Interestingly, BDNF, CNTF, and IGF-1 were markedly upregulated after NSC transplantation in DSP compared with the group of NSC in SP and the group of DSP. These neurotrophic factors may protect host cells from secondary damage and improve cognitive function [50]. Moreover, the recovery of synaptic and cognitive function, the survival and differentiation of NSC, may also be facilitated by the elevated neurotrophic factors.

However, there are some limitations to this study. First, the pathological changes of AD are very complex, and the Aβ injection model of AD could not completely reflect the progressive changes of AD. Thus, the effect of NSC transplantation in DSP should be estimated in other AD models such as Aβ injection using osmotic minipump and transgenic mice. Second, learning and memory function only was tested at 4 weeks after NSC transplantation. Therapeutic efficiency would be further examined for a prolonged period of time to make a better foundation to the clinic use.

In summary, we have demonstrated that NSC transplantation in DSP significantly improved spatial learning and memory deficits in AD rats. Our findings clearly demonstrated that NSC transplantation in DSP can improve function via both direct and bystander-like mechanisms. DSP could directly increase the survival and differentiation of NSC into neurons, decrease the neuronal apoptosis and synaptic loss, on another hand, NSC transplantation in DSP also provided a trophic support to modulate inflammation and facilitate the neuroprotection. Our data firstly provide novel evidences that NSC transplantation with DSP was more efficient to enhance neuroprotection, neurogenesis, antineuroinflammatory, and pro-neurotrophic secretion in an Aβ_1–40_-infused rat model to improve neuronal survival and differentiation and synaptic recovery, thus to prevent memory deficiency. This work profoundly supported the therapeutic potential and clinical use of NSC with biomaterials in the treatment of AD.

## Electronic supplementary material

Below is the link to the electronic supplementary material.

Fig. S1 Dose-dependent neurotoxicity of Aβ_1–40_ on NSC. Increasing concentrations of Aβ_1–40_ were added to the culture medium of cells, and the cytotoxicity was estimated after 24 h using the MTS assay. *P < 0.05 and **P < 0.01 versus the non-treated groups. (GIF 6 kb)

High resolution image (TIFF 747 kb)

Supplementary Table 1 (DOC 36 kb)
