# Supplementary figures and images for: Designer Self-Assemble Peptides Maximize the Therapeutic Benefits of Neural Stem Cell Transplantation for Alzheimer’s Disease via Enhancing Neuron Differentiation and Paracrine Action
Source: Mol Neurobiol. 2015 Jan 14;53(2):1108–23. doi: 10.1007/s12035-014-9069-y (PMC4752586; doi:10.1007/s12035-014-9069-y)

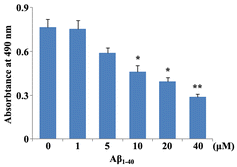

Supplement: Supplementary file 1 — Dose-dependent neurotoxicity of Aβ1–40 on NSC. Increasing concentrations of Aβ1–40 were added to the culture medium of cells, and the cytotoxicity was estimated after 24 h using the MTS assay. *P < 0.05 and **P < 0.01 versus the non-treated groups. (GIF 6 kb) [file 12035_2014_9069_Fig10_ESM.gif]

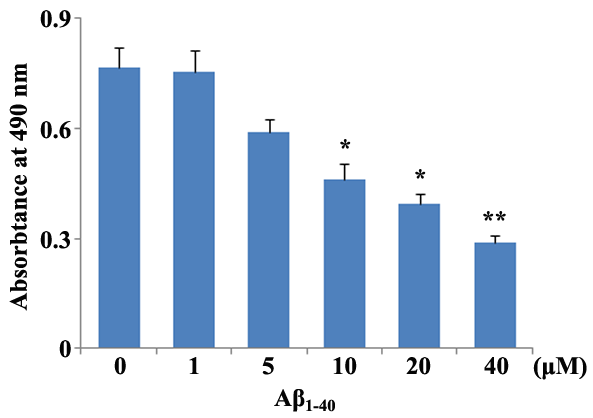

Supplement: Supplementary file 2 — High resolution image (TIFF 747 kb) [file 12035_2014_9069_MOESM1_ESM.tif]
