# Supplementary material for: Designer Self-Assemble Peptides Maximize the Therapeutic Benefits of Neural Stem Cell Transplantation for Alzheimer’s Disease via Enhancing Neuron Differentiation and Paracrine Action
Source: Mol Neurobiol. 2015 Jan 14;53(2):1108–23. doi: 10.1007/s12035-014-9069-y (PMC4752586; doi:10.1007/s12035-014-9069-y)
Supplement: Supplementary file 3 — (DOC 36 kb) [file 12035_2014_9069_MOESM2_ESM.doc]

Table 1　 The levels of cytokines in the hippocampal tissues.（pg/ml, x±s）

| Group | BDNF | CNTF | IGF-1 | TNF-α | IL-1β | IL-10 |
| --- | --- | --- | --- | --- | --- | --- |
| Normal | 682.44±80.12 | 233.64±12.45 | 143.11±9.39 | 375.29±38.25 | 211.45±11.82 | 124.54±12.46 |
| Control | 321.73±45.77 | 154.38±14.79 | 81.04±10.36 | 681.18±48.92 | 389.21±39.41 | 72.63±11.49 |
| NSC | 468.21±55.13^aa)^ | 146.92±21.27 | 89.57±10.03 | 543.76±44.31^a)^ | 312.4±30.17^a)^ | 117.98±20.41^a)^ |
| SP | 298.74±31.94 | 158.09±16.31 | 76.52±8.48 | 587.24±47.91 | 328.19±28.36 | 98.35±13.26 |
| NSC+SP | 496.33±37.41^aa)^ | 168.31±12.59 | 112.38±11.27^a)^ | 412.44±28.35^aa)^ | 298.74±32.74^a)^ | 142.66±18.35^a)^ |
| DSP | 307.89±40.85 | 138.93±19.83 | 85.32±9.05 | 600.38±51.28 | 302.94±35.21 | 100.23±15.38 |
| NSC+DSP | 583.21±78.18^aa,b,cc)^ | 208.77±19.18^a,b,c)^ | 128.32±12.28^a,c)^ | 391.48±40.21^aa,cc)^ | 245.26±20.88^aa,b,c)^ | 217.93±25.46^aa,bb,cc)^ |

^a^ *P* < 0.05 and ^aa^ *P* < 0.01 versus Control, ^b^ *P* < 0.05 and ^bb^ *P* < 0.01 versus NSC+SP, ^c^ *P* < 0.05 and ^cc^ *P* < 0.01 versus DSP.
